# A Platform for Validating Colorectal Cancer Driver Genes Using Mouse Organoids

**DOI:** 10.3389/fgene.2021.698771

**Published:** 2021-06-28

**Authors:** Haruna Takeda

**Affiliations:** Laboratory of Molecular Genetics, National Cancer Center Research Institute, Tokyo, Japan

**Keywords:** mouse organoid, colorectal cancer, validation, CRISPR-Cas9, transplantation

## Abstract

Systematic approaches for functionally validating cancer genes are needed since numerous genes mutated in cancer tissues have been identified from cancer genome sequencing. The mouse organoid culture system has been extensively used in the field of cancer research since mouse organoids can faithfully recapitulate the physiological behavior of the cells. Taking advantage of this, we recently described a platform for functionally validating colorectal cancer (CRC) driver genes that utilized CRISPR-Cas9 in mouse intestinal tumor organoids. In this review, we will describe how mouse organoids have been applied to CRC research and focus on how CRC genes can be validated using mouse organoids.

## Introduction

Recent multi-omics studies have identified numerous genes mutated or deregulated in cancer tissues (Kim et al., 2011; Cerami et al., 2012). These comprehensive datasets have provided us the genomic landscape of cancer; however, it is still difficult to enrich true cancer driver genes simply from the genomic data alone. To get a whole picture of cancer driver genes, it will be necessary to validate the function of mutated genes one by one and analyze their functions in cancer development and progression.

One of the most standard approaches for cancer gene validation is to generate genetically engineered mice (GEM) and evaluate whether the mutant allele promotes tumor development *in vivo*. However, it is time consuming to generate GEM, and usually we have to continue mouse crosses using cancer-prone mice such as *Apc* mutant mice (Moser et al., 1990; Fodde et al., 1994; Oshima et al., 1995; Shibata et al., 1997; Colnot et al., 2004) to generate compound mutant mice, since a single genetic alteration is often not sufficient to induce tumors for most of the genes. For example, in mice carrying an activating mutation of *Kras, KrasG12D*, in the epithelial cells of the small intestine and colon, the mice showed hyperplasia throughout the colonic epithelium as shown by an extreme lengthening of the crypts; however, no tumor development was observed (Haigis et al., 2008; Sakai et al., 2017). In contrast, compound mutant mice carrying *KrasG12D* and a loss of function mutation of *Apc* developed invasive adenocarcinomas in the small intestine as well as in the colon (Haigis et al., 2008; Sakai et al., 2017). Furthermore, although *TP53* mutations are observed in nearly half of late-stage human colorectal cancer (CRC), *Trp53LSL-R270H* knock-in mice infrequently developed intestinal tumors (Olive et al., 2004), while *Apc*Δ*716/*+*:Trp53LSL-R270H/*+*:Villin-CreER*^*T*2^ mice developed advanced adenocarcinomas in the small intestine and colon (Sakai et al., 2017).

The *Apc* mutant mice have been frequently used for CRC GEM models (Moser et al., 1990; Fodde et al., 1994; Oshima et al., 1995); however, the models have some limitations. They frequently develop small intestinal rather than colonic tumors, and overall tumor burden limits the time whereby malignant progression can occur (Taketo and Edelmann, 2009). Therefore, studying metastatic CRC has been hampered by a lack of mouse models that develop spontaneous metastatic colon cancers.

Another approach to validate candidate cancer genes (CCGs) that has been extensively taken so far is to use cancer cell lines. To validate tumor suppressive functions of a gene, for example, the CCG is knocked down or knocked out and transplanted to mice to evaluate the tumor-promoting function *in vivo*. However, cancer cell lines usually carry numerous genetic alterations in cancer driver genes as illustrated in the Cancer Cell Line Encyclopedia (Ghandi et al., 2019); therefore, it is often difficult to evaluate the oncogenic ability of a single gene mutation.

**Figure 1:**
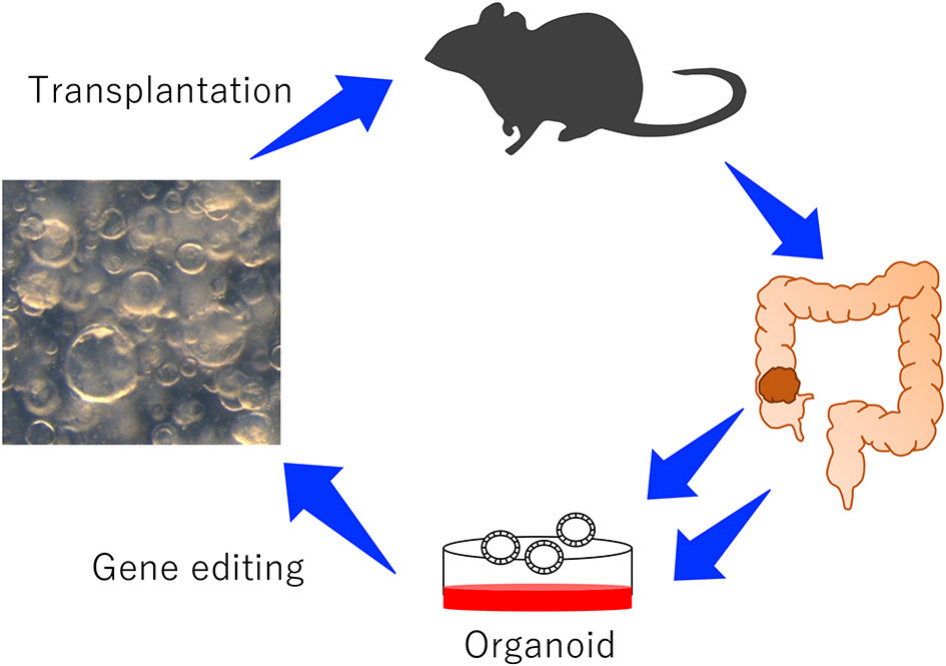
An experimental system to study the function of CRC genes using mouse-derived organoids. Colonic organoids or colonic tumor organoids were established from wild-type mice or GEM, genetically manipulated, and transplanted to mice to induce tumor development.

Recently, to validate numerous CCGs in a short period of time, new approaches which rely on transplantation of mouse organoids have been reported (Figure 1; Takeda et al., 2019). The organoid culture system was first reported in 2009 using mouse intestinal crypts (Sato et al., 2009). The culture system can reproduce long-lived, self-organizing crypt-villus organoids which retain the physiological functions of epithelial cells (Sato et al., 2011b). In addition to normal epithelial cells, tumor-derived organoids can be cultured to study the behavior of tumor cells *in vitro*. When tumor organoids are transplanted to mice, organoids can reproduce tumors which are histopathologically similar to tumors originally developed in mice or humans (Lee et al., 2018), which is one of the great advantages to use organoids. In contrast, cancer cells cultured in the plastic dish often develop tumors which are histopathologically different from tumors originally developed in mice or human.

Ideally, CCGs should be validated in the cells derived from the same tissue in which CCGs were identified, since some cancer driver genes are tissue specific. Organoids can be established from most of the tissue where solid cancer can arise, such as the small intestine (Sato et al., 2009), colon (Sato et al., 2011b), stomach (Barker et al., 2010), liver (Huch et al., 2013b), and pancreas (Greggio et al., 2013; Huch et al., 2013a). Therefore, we can validate CCGs identified from almost any solid tissues using organoids.

In this review, we will especially focus on validating colorectal (CRC) CCGs using mouse-derived organoids.

## Establishment of Mouse-Derived Organoids

The organoid culture system was first established from mouse intestinal epithelial cells. Intestinal crypts were isolated using EDTA, embedded in Matrigel and cultured in the media containing R-spondins, Noggin, and epidermal growth factor (EGF), which are known to be essential factors for intestinal stem cell maintenance (Sato et al., 2009). The next paper from the same group showed that single Lgr5 positive intestinal stem cells could reconstitute the functional crypt-villus structure *in vitro* which was composed of stem cells and differentiated cells such as goblet cells and Paneth cells (Sato et al., 2011b). The colonic crypt lacks Paneth cells which function as a stem cell niche by producing Wnt3a; therefore, the addition of Wnt3a to the culture media is necessary for the colonic organoid culture (Sato et al., 2011b). In contrast, the *Apc* deficient tumor organoids can survive in the media lacking R-spondins and Wnt3a, since tumor cells carry bi-allelic inactivation of *Apc*, which causes ligand-independent constitutive activation of Wnt signaling (Sato et al., 2011a).

Genetic manipulations of organoids can be done; delivery of plasmids or oligo DNAs or oligo RNAs to organoids can be achieved by lipofection, by electroporation, or by virus-mediated transduction (Miyoshi and Stappenbeck, 2013; Onuma et al., 2013; Drost et al., 2015; Matano et al., 2015); therefore, organoids have been widely used for understanding the physiological roles of intestinal epithelial cells or elucidating molecular mechanisms underlying intestinal diseases such as cancer. The introduction efficiency of plasmids by lentivirus can be improved by longer incubation time as described in Onuma et al. (2013); however, complete knockdown of gene expression using shRNA vectors seemed to be still difficult (Onuma et al., 2013).

One of the advantages to use mouse-derived organoids is that they are genetically defined, so that the function of a single genetic alteration can be clearly validated. In contrast, human-derived organoids show genetic variations which often hamper obtaining reproducible results between organoids from different patients.

## Mouse Models for CRC Using Mouse Organoids

Recently, the approach to model CRC development and metastasis that relies on orthotopic transplantation in mice has been reported (O'rourke et al., 2017; Roper et al., 2017). Roper et al. introduced U6::sgApc-EFS::Cas9-P2A-GFP lentivirus into wild-type colonic organoids to generate *Apc* mutant organoids, which was subsequently transplanted to the mouse colon using a colonoscopy-guided mucosal injection system. The mice developed adenomas in which nuclear β-catenin accumulation was observed. To model more advanced tumors, they generated colonic organoids carrying mutations in *Kras* and *Trp53* in addition to *Apc* then transplanted them orthotopically. The mice developed invasive adenocarcinomas as well as liver metastases. O'Rourke et al. also used a similar approach; they engineered wild-type organoids to generate mutant organoids carrying *shApc* or *shApc/KrasG12D* or *shApc/KrasG12D/p53mut* then transplanted them orthotopically. Adenomas or adenocarcinomas or metastatic adenocarcinomas were developed when *shApc* or *shApc/KrasG12D* or *shApc/KrasG12D/p53mut* were transplanted, respectively (O'rourke et al., 2017). These results were consistent with what were seen in genetically engineered mice; *Apc* konckout mice developed adenomas, whereas mice carrying an *Apc* mutation and a KrasG12D mutation developed invasive adenomas. The data clearly show that orthotopic transplantation of mouse tumor organoids can be used to model the multi-step progression model of human CRC (O'rourke et al., 2017; Roper et al., 2017) and also that these experimental systems enable rapid *in vivo* characterization of cancer-associated genes and reproduce the entire spectrum of tumor progression and metastasis.

## Mouse Organoids to Elucidate Molecular Mechanisms Underlying CRC Metastasis

To investigate the functional role of cancer stem cells in CRC development and metastasis, De Sousa E Melo et al. established an excellent system. They generated organoids established from colonic tumors developed in mice carrying mutations in *Apc, Kras, Smad4*, and *Trp53* (De Sousa E Melo et al., 2017). In addition, they introduced a diphtheria toxin receptor (DTR) fused to an enhanced green fluorescent protein (GFP) under the control of endogenous *Lgr5* so that Lgr5 positive CRC stem cells can be selectively killed by administration of diphtheria toxin. Transplantation of organoids carrying four different mutations induced metastatic tumors. In this model, depletion of Lgr5 positive CRC stem cells by the DTR system reduced the number of metastatic foci as well as the tumor volume. These data showed that Lgr5 positive CRC stem cells are required for initiation and maintenance of cancer metastasis.

To test the therapeutic effects on CRC metastasis, Tauriello et al. generated compound mutant mice carrying Lgr5-CreERT2 and four key CRC mutations: *Apcfl/fl*, *KrasLSL-G12D*, *Tgfbr2fl/fl*, and *Trp53fl/fl* (designated L, A, K, T, and P, respectively) and established organoids from tumors developed in the LAKTP mice (Tauriello et al., 2018). LAKTP organoids developed metastatic tumors when transplanted orthotopically. Using this model, they found that inhibition of TGFb signaling conferred tumors susceptible to anti-PD-1-PD-L1 therapy in CRC metastasis (Tauriello et al., 2018).

Sakai et al. showed the combination of key CRC driver mutations that conferred the metastatic ability to the cells by constructing the library for intestinal tumor organoids derived from mouse tumor carrying ApcΔ716 (A), KrasG12D (K), Tgfbr2-/-(T), and Trp53R270H (P) mutations in various combinations. The metastatic ability of organoids can be evaluated by whether organoids transplanted in the spleen can seed tumors in the liver. A and AK organoids are non-invasive non-metastatic cells, while AT and AP organoids are invasive non-metastatic cells. In contrast, AKTP cells are invasive metastatic cells (Sakai et al., 2017). Using the organoid library, the group showed that non-metastatic AT or AP cells could metastasize when transplanted with metastatic AKTP cells and could survive under the new environment after depleting metastatic AKTP cells. The data experimentally proved that the metastatic process could be promoted by polyclonal cell populations (Kok et al., 2021).

## A Platform for Functional Validation of CCGs Using CRISPR-Cas9 In Mouse Tumor Organoids

**Figure 2:**
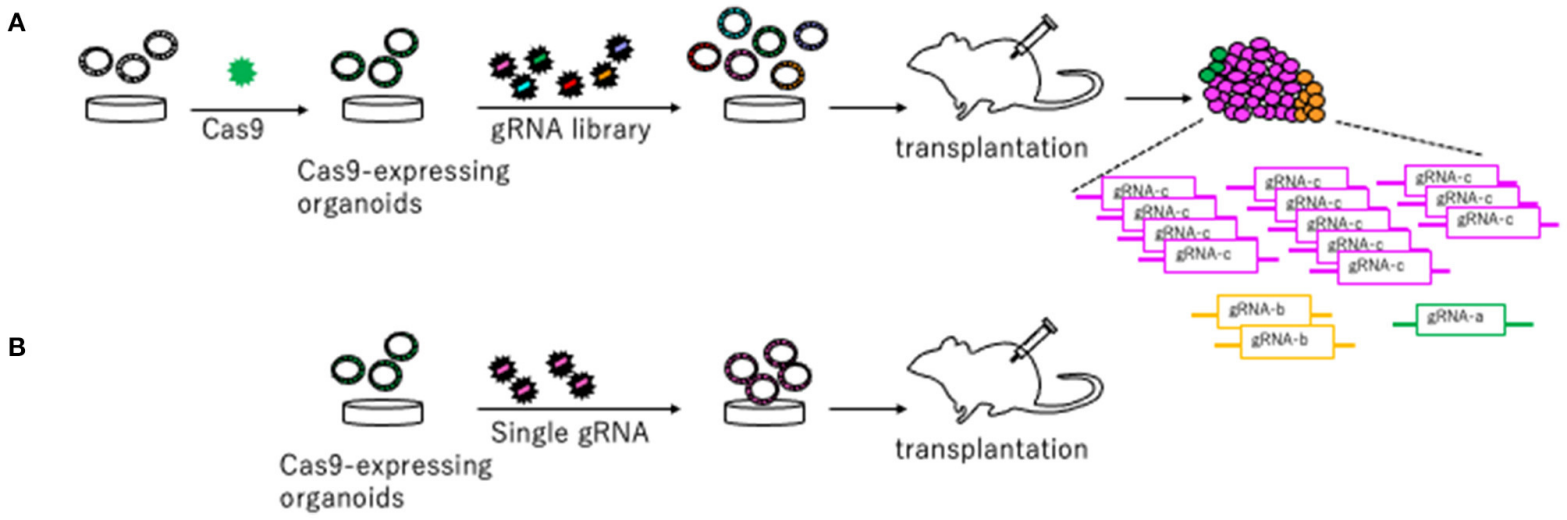
A platform to validate candidate tumor suppressor genes using CRISPR-Cas9 in mouse organoids described in Takeda et al. (2019). **(A)** Cas9 is introduced to organoids to generate Cas9-expressing organoids. The gRNA library targeting candidate tumor suppressor genes are generated and introduced to Cas9-expressing organoids. Organoids carrying the gRNA library are transplanted to mice to induce tumors. The gRNAs over-represented in the tumor tissue compared to pre-injected organoids are likely to be tumor suppressors. **(B)** Further individual validation by introducing a single gRNA targeting a gene in Cas9-expressing organoids and subsequent transplantation makes it possible to confirm a tumor suppressive function of a driver gene.

To identify novel CRC driver genes, our group performed genome-wide *Sleeping Beauty* (SB) transposon mutagenesis in mice (Takeda et al., 2015, 2021). SB transposon mutagenesis screen is a powerful tool for *in vivo* screening for CCGs in mice (Collier et al., 2005; Dupuy et al., 2005; Copeland and Jenkins, 2010). We identified 1,333 CCGs and then compared these CCGs to the datasets for genes mutated in human CRC to enrich commonly mutated genes between human and mice. These genes should be potent cancer driver genes since their functions in tumor development are conserved between species. To validate these CCGs which were commonly identified from human and mice, we established a new approach that utilized CRISPR-Cas9 in mouse tumor organoid (Takeda et al., 2019) (Figure 2). We used mouse AK organoids established from colonic tumors developed in mice carrying Villin-CreER^T2^/+, lsl-KrasG12D/+, and *Apc*Δ*716/*+ alleles (Sakai et al., 2017), since *APC* and *KRAS* mutations were observed in >80% and >40% of CRC, respectively. We first introduced Cas9 by lenti-virus and established Cas9-expressing AK organoids (Figure 2). To validate several CCGs efficiently, we generated the gRNA libraries targeting 10 different genes and introduced them to AK-Cas9 organoids to establish the mixture of organoids carrying mutations in 10 different genes. We used the sequence of gRNAs published in Koike-Yusa et al. (2014) and Tzelepis et al. (2016). The organoids were subsequently transplanted subcutaneously to induce tumor development (Figure 2). AK-Cas9 organoids alone rarely induced tumor development when transplanted, while AK-Cas9 carrying the gRNA mixture developed tumors. Analysis of over-represented gRNAs in tumor genomes identified responsible genes for tumor development (Figure 2). Next, we validated the genes individually by the introduction of a single gRNA targeting a candidate gene to AK-Cas9 organoids and transplantation to mice. To validate CCGs by the CRISPR-Cas9 system, it is necessary to use more than one gRNAs to confirm that the results are not biased by the off-target effect. These analyses showed that *Acvr1b, Acvr2a*, and *Arid2* were colorectal tumor suppressors. *Acvr1b* and *Acvr2a* encode activin receptor type Ib and type IIa, respectively, which belong to the TGF-β superfamily. *In silico* analyses using The Cancer Genome Atlas (TCGA) dataset of genes mutated in CRC (TCGA, 2012) showed that mutations in TGF-β receptor type II and activin receptors co-occurred. To functionally validate the cooperative function between receptors for TGF-β and activin, we knocked out activin receptors by CRISPR-Cas9 in organoids carrying mutations in *Apc, Kras*, and *Tgfbr2*, which encodes TGF-β receptor type II and is then transplanted to mice. The efficiency for tumor development was accelerated when activin receptors were knocked out, showing that receptors for TGF-β and activin synergistically function in tumor suppression. These data showed that the mouse organoid culture system is a powerful platform for validating CCGs and elucidating the molecular mechanisms underlying cancer development.

## Discussion

The mouse organoid has been extensively used in the biomedical research field since it was first reported. The organoid culture has enabled us to assess the reponse of intestinal epithelial cells to anti-cancer drugs, growth factors, or inflammatory cytokines and analyze the detailed molecular mechanisms *in vitro* (Lindemans et al., 2015; De Sousa E Melo et al., 2017; Kajino-Sakamoto et al., 2021). A recent study has performed a genome-scale CRISPR screen to identify genes that regulate Wnt-dependent renewal of gastric epithelial cells and identified *Alk, Bclaf3*, and *Prkra* as Wnt pathway regulators (Murakami et al., 2021); the data show that mouse organoids are also useful for stem cell research.

Studies on metastatic CRC have been hampered by a lack of GEM models. The approach described in this article which relies on transplantation of genetically engineered organoids is definitely a powerful tool to model metastatic CRC. The approach will make it possible to validate the metastatic ability of various combinations of CRC driver genes and to establish new models for CRC metastasis. Further studies to elucidate the molecular mechanisms promoting metastasis will definitely help accelerate the development of new therapeutic strategies.

## Author Contributions

The author confirms being the sole contributor of this work and has approved it for publication.

## Conflict of Interest

The author declares that the research was conducted in the absence of any commercial or financial relationships that could be construed as a potential conflict of interest.
